# The effect of control measures on COVID-19 transmission in South Korea

**DOI:** 10.1371/journal.pone.0249262

**Published:** 2021-03-29

**Authors:** Taeyong Lee, Hee-Dae Kwon, Jeehyun Lee

**Affiliations:** 1 School of Mathematics and Computing (Mathematics), Yonsei University, Seoul, Korea; 2 Department of Mathematics, Inha University, Incheon, Korea; 3 School of Mathematics and Computing, Yonsei University, Seoul, Korea; Nanyang Technological University, SINGAPORE

## Abstract

Countries around the world have taken control measures to mitigate the spread of COVID-19, including Korea. Social distancing is considered an essential strategy to reduce transmission in the absence of vaccination or treatment. While interventions have been successful in controlling COVID-19 in Korea, maintaining the current restrictions incurs great social costs. Thus, it is important to analyze the impact of different polices on the spread of the epidemic. To model the COVID-19 outbreak, we use an extended age-structured SEIR model with quarantine and isolation compartments. The model is calibrated to age-specific cumulative confirmed cases provided by the Korea Disease Control and Prevention Agency (KDCA). Four control measures—school closure, social distancing, quarantine, and isolation—are investigated. Because the infectiousness of the exposed has been controversial, we study two major scenarios, considering contributions to infection of the exposed, the quarantined, and the isolated. Assuming the transmission rate would increase more than 1.7 times after the end of social distancing, a second outbreak is expected in the first scenario. The epidemic threshold for increase of contacts between teenagers after school reopening is 3.3 times, which brings the net reproduction number to 1. The threshold values are higher in the second scenario. If the average time taken until isolation and quarantine reduces from three days to two, cumulative cases are reduced by 60% and 47% in the first scenario, respectively. Meanwhile, the reduction is 33% and 41%, respectively, for rapid isolation and quarantine in the second scenario. Without social distancing, a second wave is possible, irrespective of whether we assume risk of infection by the exposed. In the non-infectivity of the exposed scenario, early detection and isolation are significantly more effective than quarantine. Furthermore, quarantining the exposed is as important as isolating the infectious when we assume that the exposed also contribute to infection.

## Introduction

The 2019 novel coronavirus (COVID-19) has spread rapidly worldwide since the first case was reported in Hubei, Wuhan, China in December 2019. By 11 March 2020, more than 100,000 cases had been identified globally, and the World Health Organization (WHO) declared the situation a pandemic. Many countries and regions have taken control measures to mitigate and slow the spread of the virus. It is important to analyze whether these measures are efficient in controlling the epidemic.

Several studies have predicted the dynamics of diseases and assessed strategies to combat them. In 1972, Kermack and McKendrick developed the susceptible-infective-recovered (SIR) model, a simple and basic compartment model to describe an epidemic [1]. Hethcote and others investigated deterministic epidemiological models and studied several features of modified models such as SEIR and SLAIR [2–4]. In the wake of COVID-19, many researchers have attempted to make epidemic predictions and evaluation of control measures based on mathematical modeling and simulation.

A time delay dynamic system with external sources has been proposed to explain the trend of the disease’s spread in China [5]. Jia et al. [6] analysed the migration effect and impact of policy with provinces in China. In another recent study [7], the authors developed a new *θ*-SEIHRD model for coronavirus transmission reflecting undetected infections. Lin et al. considered a conceptual model for the COVID-19 outbreak in Wuhan, taking individual behavioral responses and government actions into account where the transmission rate function introduced in He et el. [8] is employed [9]. The potential effects of delay of school opening on the COVID-19 outbreak in South Korea has also been explored through mathematical modeling [10]. However the model limits its interest on school closure only considering two age groups (under and over 19).

This study predicts epidemics dynamics and estimates the effects of policies on the spread of COVID-19 in South Korea. To model the COVID-19 outbreak, we use an age-structured SEIR model with quarantine and isolation compartments. It divides the population into nine age groups that are consistent with the daily reports of cumulative confirmed cases provided by the Korea Disease Control and Prevention Agency & Prevention (KDCA). The model allows us to predict the disease dynamics and evaluate different mitigation strategies. We analyze the effects of school closure and social distancing by varying the reduced level of transmission and timing of implementation. The impact of quarantine and isolation is also investigated with different coverage rates.

## Methods

### Model structure

**Fig 1:**
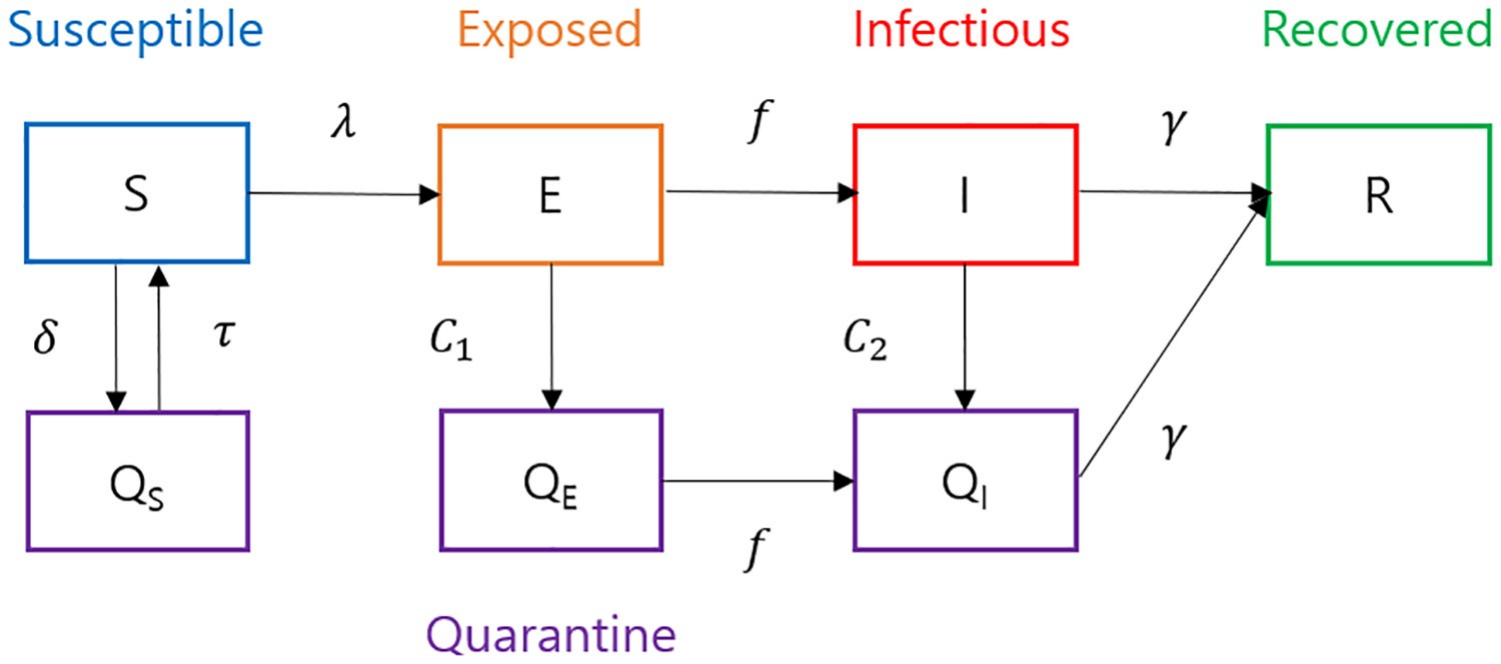
Model diagram. This figure shows the proposed model to describe the dynamics of COVID-19 in South Korea, based on the SEIR model. Three compartments are added to represent the quarantined susceptible *Q*_*S*_, quarantined exposed *Q*_*E*_, and isolated *Q*_*I*_.

To analyze the effect of control measures on the spread of COVID-19 in South Korea, we extend the deterministic SEIR model as shown in Fig 1 and the corresponding system of equations is given in S1 Appendix. The basic compartments *S*, *E*, *I*, and *R* represent the susceptible, exposed, infectious, and recovered, respectively [11]. To observe how quarantine and isolation influence the dynamics, *Q*_*S*_, *Q*_*E*_ and *Q*_*I*_ are added. Here, *Q*_*S*_ and *Q*_*E*_ indicate the quarantined susceptible and quarantined exposed through contact tracing of the confirmed cases, and *Q*_*I*_ represent those isolated due to diagnosis. The parameters used for the disease progression are described in the following section.

### Data and parameter estimation

**Fig 2:**
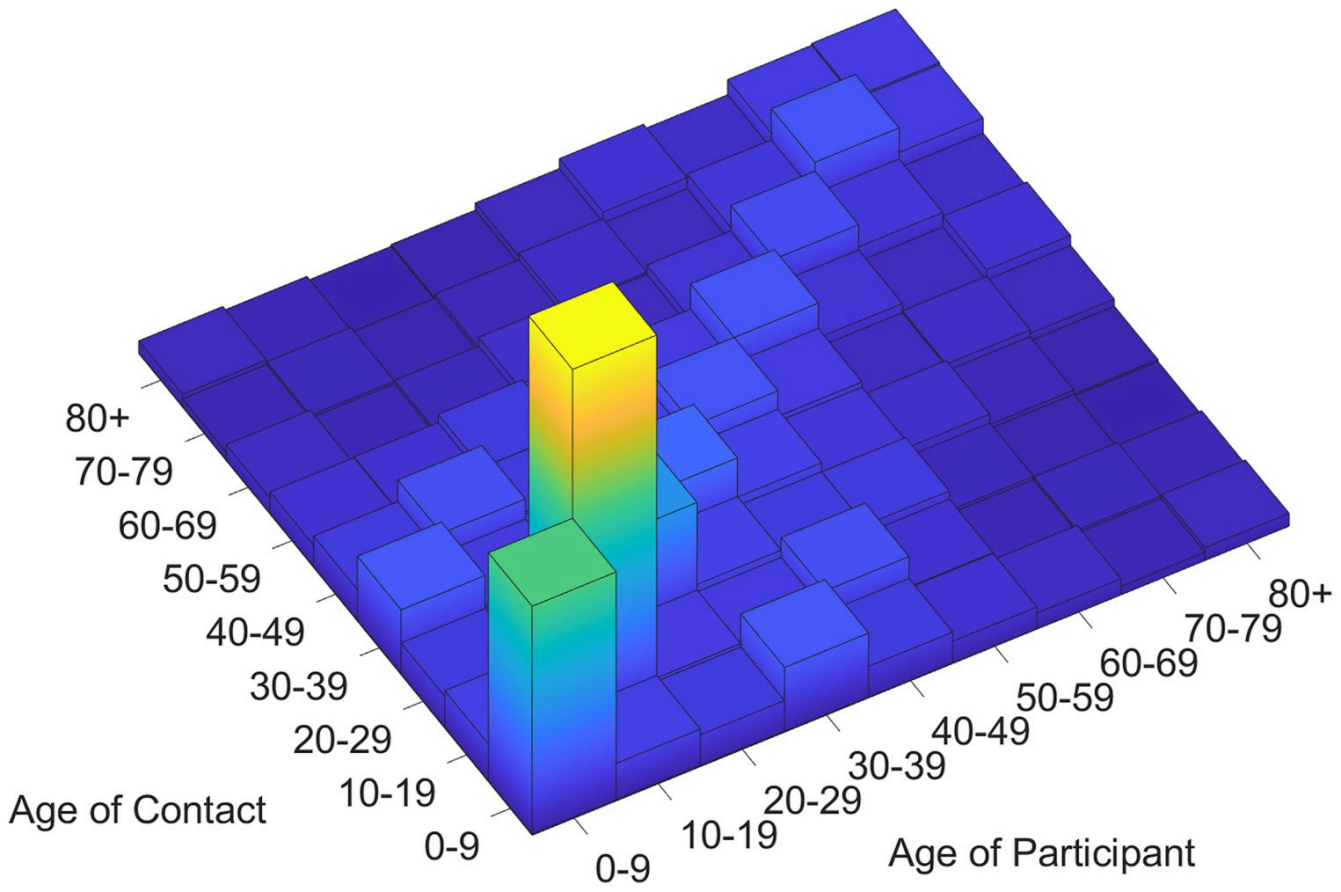
Contact rate matrix. The contact rate matrix is obtained from the POLYMOD survey data [13].

The fatality rate varies significantly by age groups, and the confirmed cases of a certain age is unusually high in South Korea are unusually high due to the outbreak being traced to a religious group called Shincheonji. Therefore, we use an age-structured model where each compartment is stratified by age groups. The Korea Disease Control and Prevention Agency & Prevention (KDCA) provides daily reports, including the number of cumulative confirmed cases. It divides the population into nine age groups: 0 − 9 years, 10 − 19 years, …, 60 − 69 years, 70 − 79 years, and older than 80 years [12]. For convenience, KDCA uses the above age structure when it is necessary to consider age-specific features. The contact information is obtained from the POLYMOD project [13], as there is no contact information survey available in South Korea. The contact rate matrix is obtained by processing the survey data to meet the reciprocity, as displayed in Fig 2 [11].

We assume that the WAIFW (Who-Acquire-Infection-From-Whom) matrix, *W*, in Eq (1), is proportional to the contact rate matrix in Fig 2. In particular, proportional factors are used to calibrate susceptibility of each age group to age-specific incidence data.

$$\begin{array}{c} W=[\begin{array}{cccc} q_{0}\left(t\right)c_{00} & q_{0}\left(t\right)c_{01} & \cdots & q_{0}\left(t\right)c_{08}\\ q_{1}\left(t\right)c_{10} & q_{1}\left(t\right)c_{11} & \cdots & q_{1}\left(t\right)c_{18}\\ \vdots & \vdots & \ddots & \vdots \\ q_{8}\left(t\right)c_{80} & q_{8}\left(t\right)c_{81} & \cdots & q_{8}\left(t\right)c_{88} \end{array}] \end{array}$$ (1)

where *c*_*ij*_ is the contact rate from the *j*th age group to the *i*th one.

**Fig 3:**
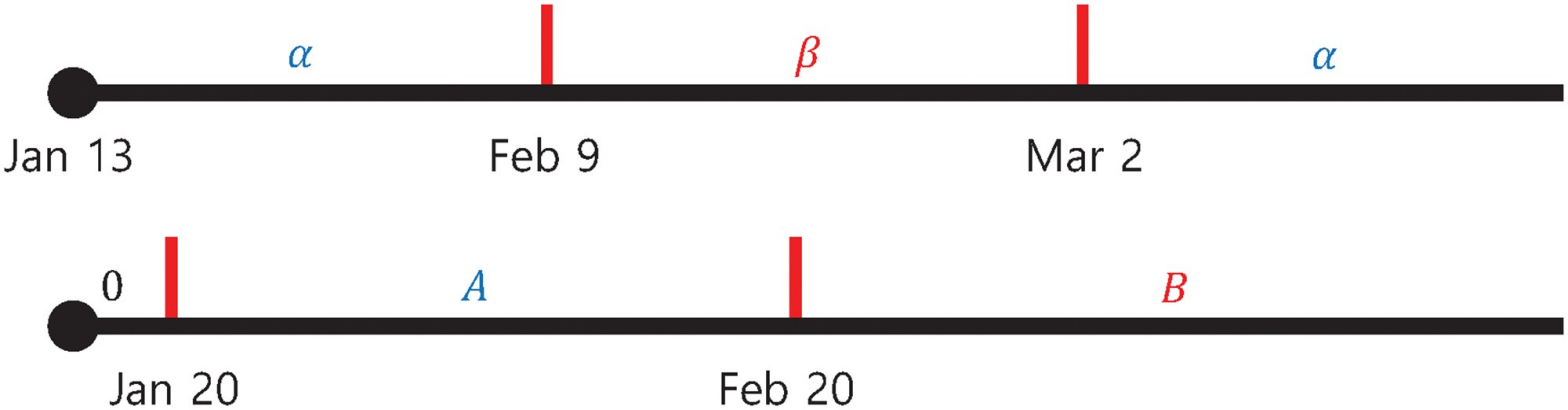
The period of piece-wise constant parameters according to the timeline of events. *q* represents the transmission rates, *q*_*i*_s in Eq (1), and *C* represents both *C*_1_ and *C*_2_. Of note, interventions began on 20 January 2020 when the first COVID-19 patient was confirmed in South Korea.

Moreover, piece-wise constant functions, *q*_*i*_(*t*), (*i* = 0, 1, …, 8) are introduced in Eq (1) to reflect the major events related to Shincheonji that contributed to the explosive outbreak in the city of Daegu and Gyeongsangbuk-do. Regarding the timeline of events, a 61-year-old woman later confirmed with COVID-19 on 18 February 2020, attended worship services on 9 and 16 February at the Shincheonji Church in Daegu, which were attended by at least 1,000 other members [14]. The terminal time of duration is chosen as of 2 March 2020, because the day marked the end of intensive testing administered to the people in her religious group. As shown in Fig 3, it is assumed that the piece-wise constant functions, *q*_*i*_(*t*), are given by

$$\begin{array}{c} q_{i}\left(t\right)=\{\begin{array}{cc} \alpha_{i}, & \text{for}\;\text{Jan.}\;\text{13}\leq t<\text{Feb.}\;\text{9}\\ \beta_{i}, & \text{for}\;\text{Feb.}\;\text{9}\leq t<\text{Mar.}\;\text{2}\\ \alpha_{i}, & \text{for}\;\text{Mar.}\;\text{2}\leq t \end{array} \end{array}$$ (2)

for *i* = 0, 1, …, 8. Here we assume *α*_*i*_ ≤ *β*_*i*_ for each *i*.

Using the WAIFW matrix, we formulate the risk of susceptible groups being infected, which is essential in analyzing compartmental models for infectious diseases [11, 15]. The force of infection (FOI) for the ith age group is defined by

$$\begin{array}{c} \lambda_{i}=\sum_{j=0}^{8}W_{ij}\left(I_{j}+\varepsilon_{E}E_{j}+\varepsilon_{Q_{E}}{Q_{E}}_{j}+\varepsilon_{Q_{I}}{Q_{I}}_{j}\right), \end{array}$$ (3)

where the subscripts *i* and *j* denote the *i*th and *j*th age group, respectively. There is no conclusive evidence on whether the exposed, *E*, are infectious [16, 17] and on the contribution level of the quarantined, *Q*_*E*_, and the isolated, *Q*_*I*_ to the FOI. Therefore, the parameter *ε* is added to incorporate the reduced infectivity of these compartments into the model. We set the baseline values of all *ε* in Eq (3) as 0 (*ε* = 0 case) and perform a sensitivity analysis considering nonzero values (*ε* ≠ 0 case).

Tracing the movements of confirmed cases, the susceptible and exposed are quarantined to move to the compartments *Q*_*S*_ and *Q*_*E*_, respectively. We assume that about 1% of the susceptible population have been quarantined cumulatively and dismissed after 14 days unless diagnosed, which determines *δ* and *τ*. The rate of quarantine from *E* to *Q*_*E*_ is denoted by *C*_1_ and the rate of isolation from *I* to *Q*_*I*_ is denoted by *C*_2_, which began from 20 January 2020. Therefore, there’s no measure before that day. Similar to transmission rates, piece-wise constants *C*_1_ and *C*_2_ are used to take into account self-quarantine and active diagnosis efforts due to the fact that the aforementioned 61-year-old woman was diagnosed as patient 31 on 20 February 2020 (Fig 3). So we assume that *C*_1_ and *C*_2_ are the piece-wise constant functions in the form of

$$\begin{array}{c} C_{i}\left(t\right)=\{\begin{array}{cc} 0, & \text{for}\;\text{Jan.}\;\text{13}\leq t<\text{Jan.}\;\text{20}\\ A_{i}, & \text{for}\;\text{Jan.}\;\text{20}\leq t<\text{Feb.}\;\text{20}\\ B_{i}, & \text{for}\;\text{Feb.}\;\text{20}\leq t \end{array} \end{array}$$ (4)

for *i* = 1, 2 where *A*_*i*_ ≤ *B*_*i*_.

We review previous studies for the pre-infectious period and infectious period [18–21]. The average values for these parameters are employed to set the pre-infectious period as 5.2 days and infectious period as 7 days, which are reciprocal of *f* and *γ*, respectively. We estimate the transmission rate *q*_*i*_(*t*) in the WAIFW matrix, quarantine rate *C*_1_(*t*), and isolation rate *C*_2_(*t*). Calibrating the values *α*_*i*_, *β*_*i*_, *A*_*i*_, and *B*_*i*_ to newly confirmed cases during the period from 20 January 2020 to 2 April 2020, we find the best-describing model for the dynamics of COVID-19 in Korea. To do this, we assume that the data are sampled from a Poisson distribution and use a maximum likelihood estimation that maximizes the likelihood function over the parameter space A consisting of non-negative real numbers. That is, we find the parameter values $\hat{\theta }=\left[{\hat{\alpha }}_{0},\ldots ,{\hat{\alpha }}_{8},{\hat{\beta }}_{0},\ldots ,{\hat{\beta }}_{8},{\hat{A}}_{1},{\hat{A}}_{2},{\hat{B}}_{1},{\hat{B}}_{2}\right]$ such that

$$\begin{array}{c} \hat{\theta }={\text{argmax}}_{\theta \in A}\{\Pi_{j}\text{Pr}\left(X_{j}=x_{j}|X_{j}∼\text{Poisson}\left(\lambda_{j}\left(\theta \right)\right)\right)\} \end{array}$$ (5)

where *X*_*j*_ and λ_*j*_ represents the data and the mean, respectively. Note that, the missing information of age on 20 February 2020 is linearly interpolated using the age proportion of the data on the day before (19 February) and the day after (21 February). The resulting parameters are summarized in the S1 Table.

**Fig 4:**
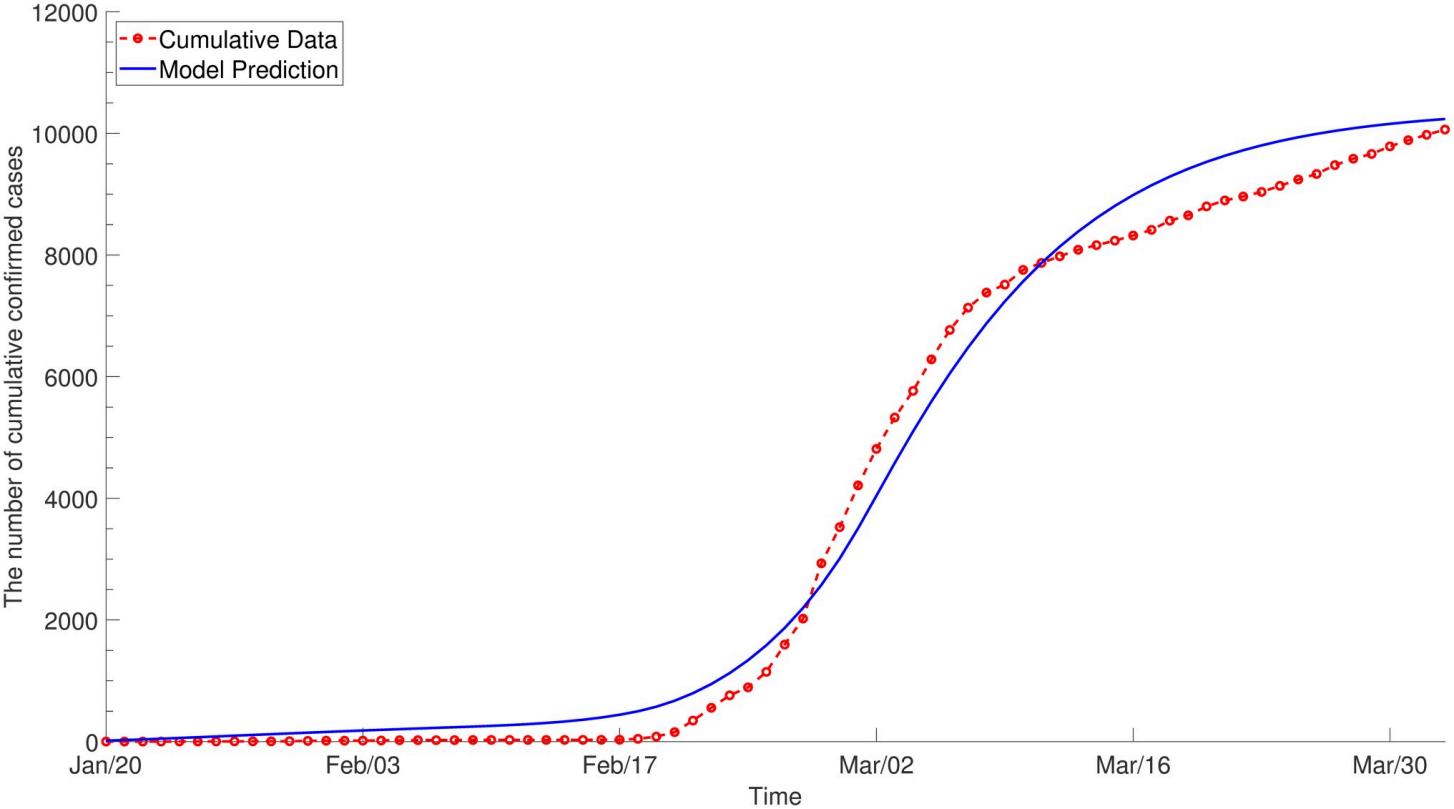
The results of parameter estimation (*ε* = 0). WAIFW and the rates of quarantine and isolation are fitted to the age-specific cumulative confirmed cases by assuming that only the infectious contribute to the force of infection. The dotted line with circles denotes the target data and the solid line denotes the model prediction with estimated parameters.

**Fig 5:**
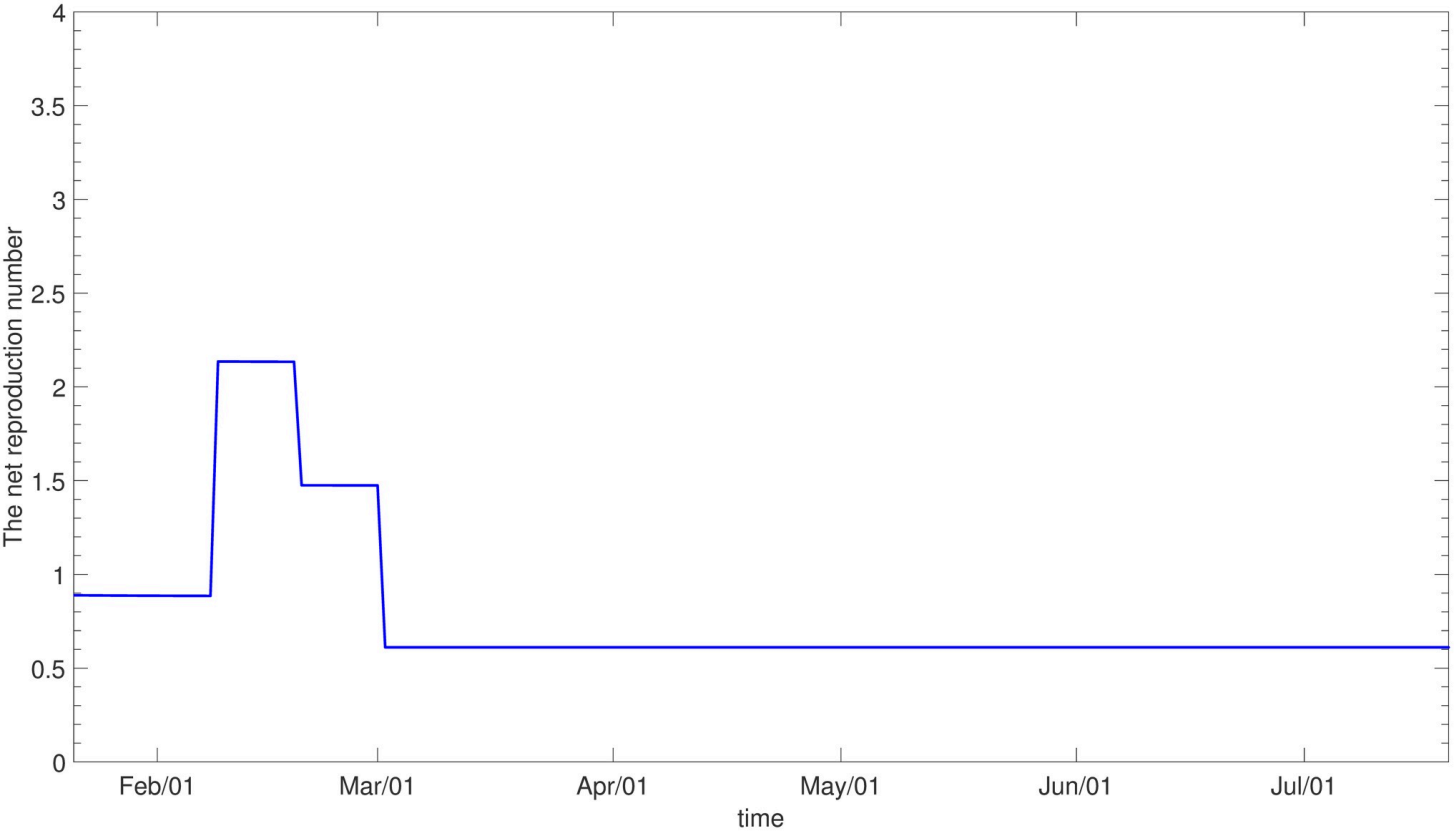
The net reproduction number (*ε* = 0).

The result of calibration assuming *ε* = 0 is displayed in Fig 4 for the whole population and the details of fitting for each age group are provided in S1 Fig. Fig 5 illustrates the estimated net reproduction number, which has been maintained at less than 1 since March due to social distancing. The estimated values for *C*_1_ and *C*_2_ are given in S2 Table.

**Fig 6:**
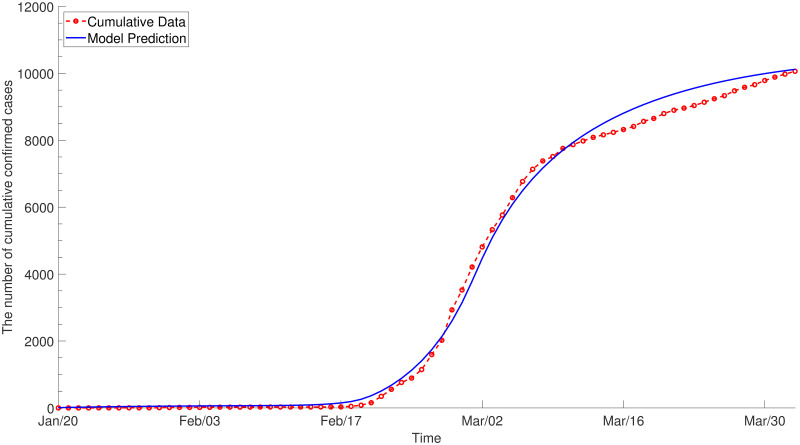
The results of parameter estimation (*ε* ≠ 0). WAIFW and the rates of quarantine and isolation are fitted to the age-specific cumulative confirmed cases by assuming reduced infectivity of the exposed, quarantined and isolated. The dotted line with circles denotes the target data and the solid line denotes the model prediction with estimated parameters.

**Fig 7:**
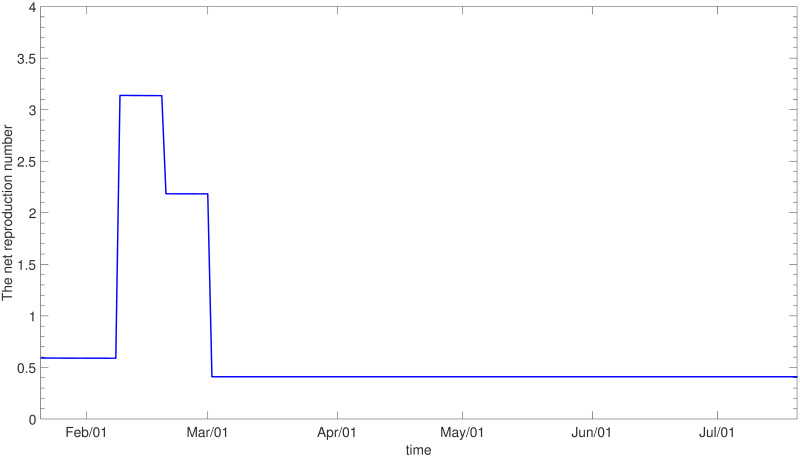
The net reproduction number (*ε* ≠ 0).

We also calibrate the model assuming *ε* ≠ 0 in Eq (3), which means the exposed, quarantined and isolated may contribute to the force of infection at a certain level [16, 17]. We assume reduced infectivity of these groups by imposing *ε*_*E*_ = 0.1, $\varepsilon_{Q_{E}}=0.01$ and $\varepsilon_{Q_{I}}=0.05$, respectively. Fig 6 shows the result of parameter estimation for the total population when *ε* ≠ 0. Age-specific results and the estimated values of *C*_1_ and *C*_2_ are given in S3 Fig and S3 Table, respectively. The corresponding net reproduction numbers are displayed in Fig 7. We observe that the dynamics here are similar to the case of *ε* = 0, while the impact of control measures may be different.

We use the 2020 expected population statistics from the Korean Statistical Information Service (KOSIS [22]) as the initial number of susceptible compartments for each age group. Seven exposed and one infectious cases are introduced at the initial state to start the simulation on 13 January 2020 as shown in Table 1, running until 20 January 2022.

**Table 1:** The initial states in the model.

Age groups	Susceptible	Exposed	Infectious	Recovered
**0-9**	2020 Population Korean	0	0	0
**10-19**		0	0	0
**20-29**		1	0	0
**30-39**		1	1	0
**40-49**		1	0	0
**50-59**		1	0	0
**60-69**		1	0	0
**70-79**		1	0	0
**80+**		1	0	0

### Control measures

#### School closure and social distancing

School closure is considered one of the most effective ways to mediate disease transmission because it is well known that the contact rate is much higher in the school-age group than in others. Most educational institutes in South Korea including elementary, middle, and high schools and colleges delayed resumption of classes and have meanwhile started on-line classes. This imposes a burden on society, triggering various issues. Thus, it is important to evaluate the impact of school closure using the age-stratified model. While it is not feasible to quantify the change in transmission due to the school closure in the current situation, several studies have reported that the rate of contact decreases by 25%∼75% during the school break [23, 24].

Another control measure of research interest is social distancing. As the number of confirmed cases decrease and social distancing continues, people’s fear and apprehensions reduce and they tend to relax adherence to suggested precautions. It is difficult to calculate the level of contact reduction brought about by school closure and social distancing. For this reason, we analyze different scenarios by varying the reduced level of transmission and timing for implementation of these measures.

#### Quarantine and isolation

In late January 2020, South Korea implemented control measures such as quarantine and isolation through tracing and diagnosis. The close contacts of confirmed cases who may be classified as susceptible or exposed are subjected to quarantine. Infectious or quarantined exposed individuals are isolated once diagnosed as COVID-19 positive. In South Korea, active and extensive laboratory tests have been performed to diagnose the reported cases. Here, we analyze the impact of quarantine and isolation on epidemic incidence by varying the rates of these measures. A relative change in rates could be achieved through time taken for coverage as well as the level of coverage.

## Results

The model calibrated to the reported confirmed cases is simulated to predict the spread of COVID-19 and evaluate the effect of control measures. As there is ambiguity regarding whether the exposed are infectious, sensitivity analysis is conducted considering two scenarios. First, we assume that the infectious is the only compartment which contributes to the force of infection (Fig 4). The second scenario assumes reduced risk of infection by the exposed, quarantined and isolated (*ε*_*E*_ = 0.1, $\varepsilon_{Q_{E}}=0.01$ and $\varepsilon_{Q_{I}}=0.05$, respectively in Fig 6).

### Prediction

**Fig 8:**
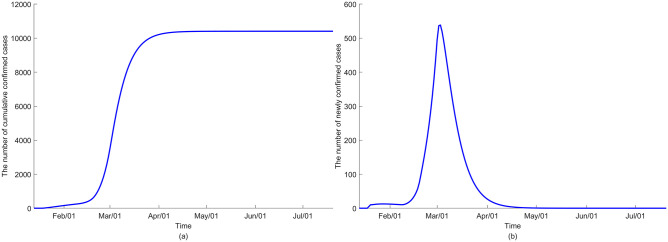
The dynamics of COVID-19 transmission under the assumption that only the infectious contribute to the FOI (*ε* = 0) in (a) cumulative confirmed cases and (b) newly confirmed cases.

**Fig 9:**
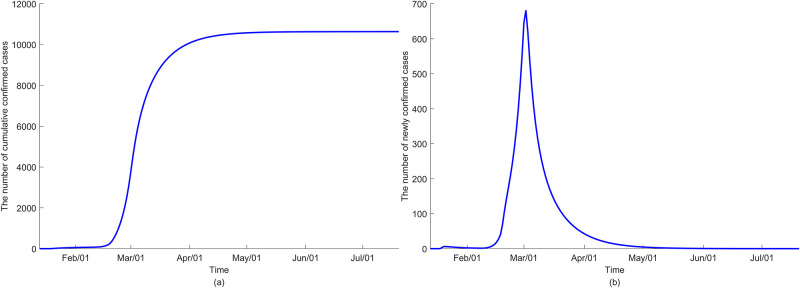
The dynamics of COVID-19 transmission under the assumption that the exposed, quarantined and isolated also contribute to the FOI (*ε* ≠ 0) in (a) cumulative confirmed cases and (b) newly confirmed cases.

Initially, we assume no changes in the control measures such as school closure, social distancing quarantine, and isolation to simulate the status quo. Fig 8 illustrates the dynamic of COVID-19 transmission assuming only the infectious contribute to infection. The model indicates that the outbreak ends around May or June after reaching the peak in early March. Meanwhile, the model output using the second scenario shows no noticeable differences in the epidemic trajectory (Fig 9). The prediction by age groups is reported in S2 and S4 Figs.

### School closure and social distancing

While the model indicates that transmissibility reduction interventions are successful in bringing the reproduction number below the epidemic threshold, maintaining restrictions at the current level imposes great social costs. The opening of schools has been delayed several times since March, and most educational institutes in South Korea are currently holding online classes. This measure has been promoted simultaneously with emphasizing the importance of personal hygiene such as wearing masks, washing hand and abstaining from gathering. Social distancing, which includes school closure, is considered an essential strategy to mediate disease transmission under the current circumstances, when neither vaccination nor treatment is available. While both social distancing and school closure are of great interest, it is difficult to calculate the level of contact reduction brought about by these measures. Therefore, we simulate different scenarios to analyse the impact of school closure and social distancing and to suggest appropriate guidelines.

To investigate the effect of school closure and social distancing by varying the reduced level of transmission, we first calculate the threshold for epidemics as shown in Table 2. Assuming that school started at 4 May 2020, when the epidemics seemed to subside, we determine the change in contact between teenagers, yielding a net reproduction number of 1. similarly, the threshold is obtained for social distancing by calibrating the scalar factor multiplied to the contact rate matrix. The threshold values for the proportional factor imposed by school closure and social distancing assuming that only the infectious contribute to the FOI, are 3.3 and 1.7, respectively. In other words, no second wave of disease spread is expected as long as the increase in transmission between teenagers stays at less than 3.3 times after school starts, assuming that only the infectious contribute to FOI. We draw a similar conclusion if the change in contact is at most 1.7 times higher than the current level without social distancing.

**Table 2:** Threshold values for the proportional factor multiplied to the contact rate.

Control measure	school closure	social distancing
*ε* = 0		
**Threshold for *R*_*n*_ = 1**	3.3	1.7
*ε* ≠ 0		
**Threshold for *R*_*n*_ = 1**	6	2.6

They yield a net reproduction number of 1 by removing each control measure.

While it is not feasible to quantify the change in transmission due to school closure in the current situation, studies have reported that contact decreases by 25%∼75% during the break, which is equivalent to the scalar factor in the range of 1.3 4. In addition, a recent research in United Kingdom found a 73% reduction in the average daily number of contacts per participant corresponding to 3.7 [25]. Combining our model output with this knowledge, we should be concerned about a second epidemic wave, because the proportional factor exceeds the threshold, which brings the net reproduction number to above 1 [11, 26]. According to the indicators in Table 2, the threshold values for the proportional factor is much higher if we assume that the exposed, quarantined and isolated as well as the infectious, can transmit the disease. However, the increase in the number of contacts due to the end of social distancing is still above the threshold in this scenario.

**Fig 10:**
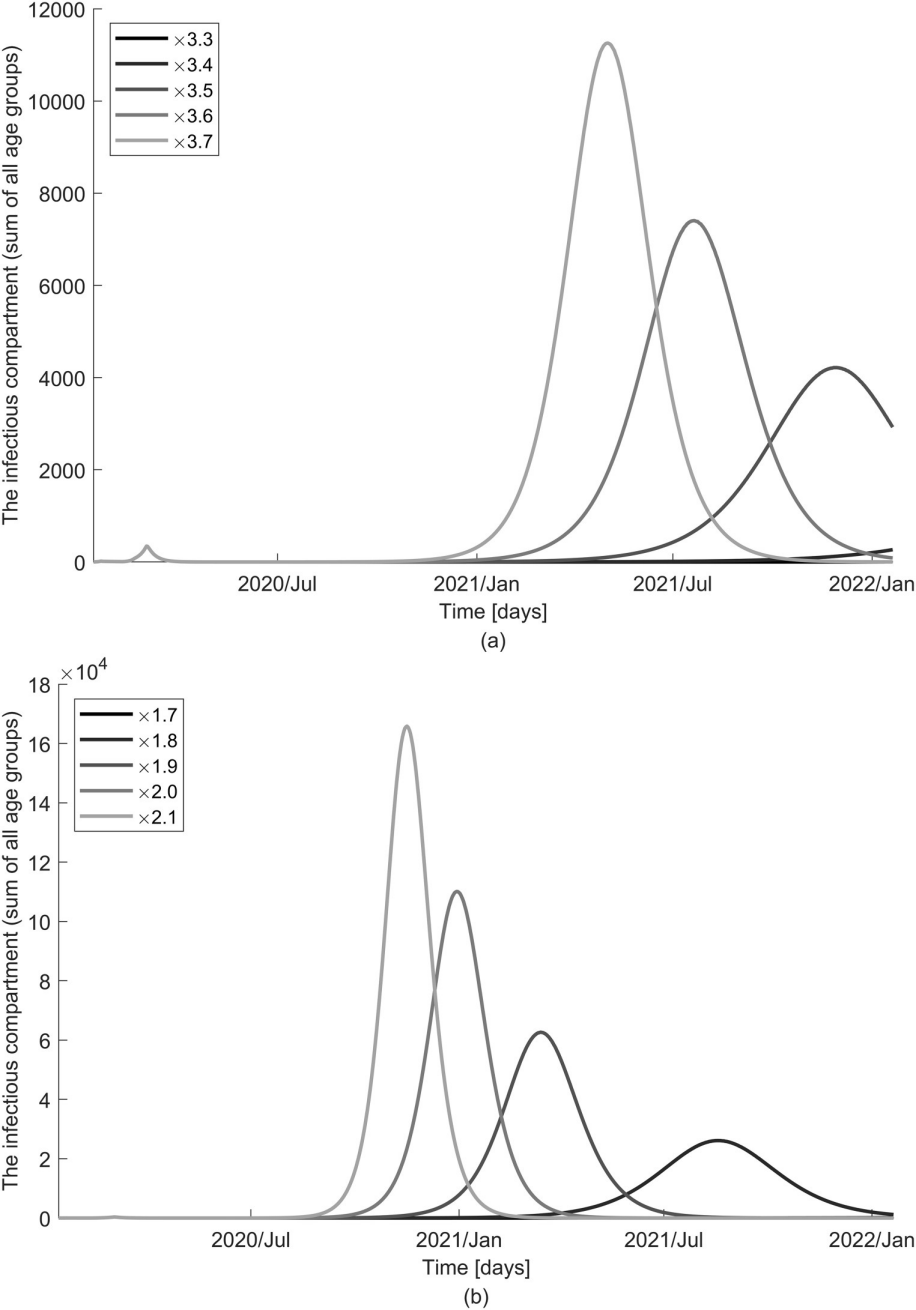
Impact of school closure and social distancing on the dynamics of disease spread when no infectivity of the exposed is assumed (*ε* = 0). The number of the infectious is displayed with various contact rates resulting from changes in control measures: (a) school starts on 4 May 2020 and (b) social distancing is relaxed on 4 May 2020.

**Fig 11:**
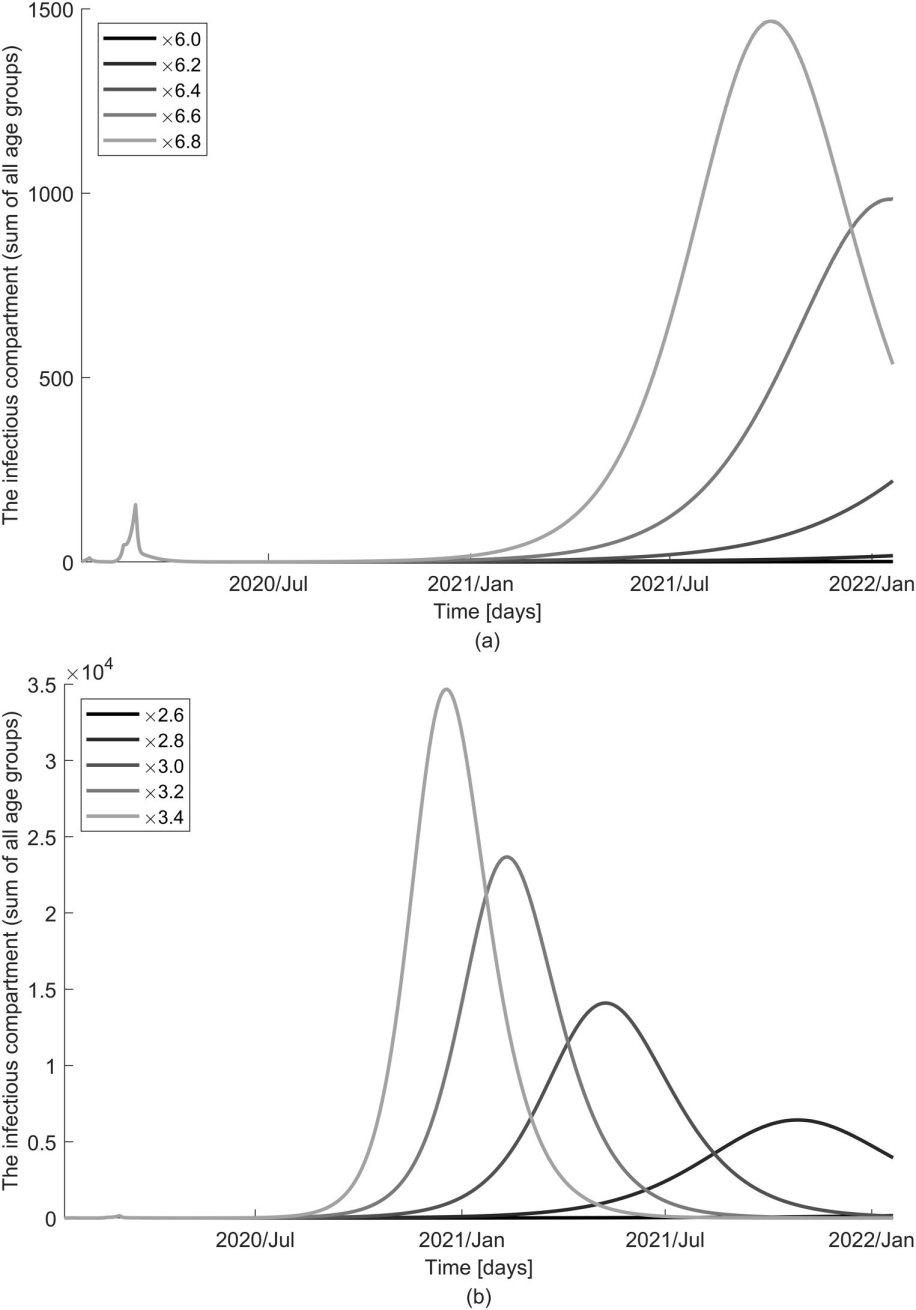
Impact of school closure and social distancing on the dynamics of disease spread when infectivity of the exposed is assumed (*ε* ≠ 0). The number of the infectious is displayed with various contact rates resulting from changes in control measures: (a) school starts on 4 May 2020 and (b) social distancing is relaxed on 4 May 2020.

The number of infectious individuals, when the reproduction number is above the epidemic threshold and no infectivity of the exposed is assumed is visualized in Fig 10. The second COVID-19 outbreak with a higher peak at an earlier stage will begin as contacts increase. The impact of school closure is relatively limited compared to that of social distancing because it is limited to only one age group. While the overall trend is similar assuming the presence of infection by the exposed, the model predicts further delayed epidemic effects with a smaller peak (Fig 11). It is noteworthy that the prediction of disease spread under different assumptions on the FOI is consistent, but the impact of control measures on the prevalence of infections is sensitive to changes. Of note, when *R*_*n*_ is slightly higher than 1, the second wave is not seen clearly on the graph because it occurs much later from the current time. We also vary the timing of school reopening and end of social distancing and find that extension by only a week causes up to a month’s delay in peak time. The results of different timings are indicated in S5–S8 Figs.

### Effect of quarantine and isolation

South Korea began conducting contact tracing in the early stages to ensure the close contacts of confirmed cases are quarantined. It is believed that reduction in transmissibility was achieved through early detection and isolation of cases due to active and extensive laboratory tests. In this section, we analyze the impact of quarantine and isolation on the disease dynamics with different rates of these measures, *C*_1_ and *C*_2_ (Fig 1). The change in rates could be attained by varying the time taken for coverage as well as the level of coverage, because *C*_1_ and *C*_2_ represent proportion of coverage during certain periods.

**Fig 12:**
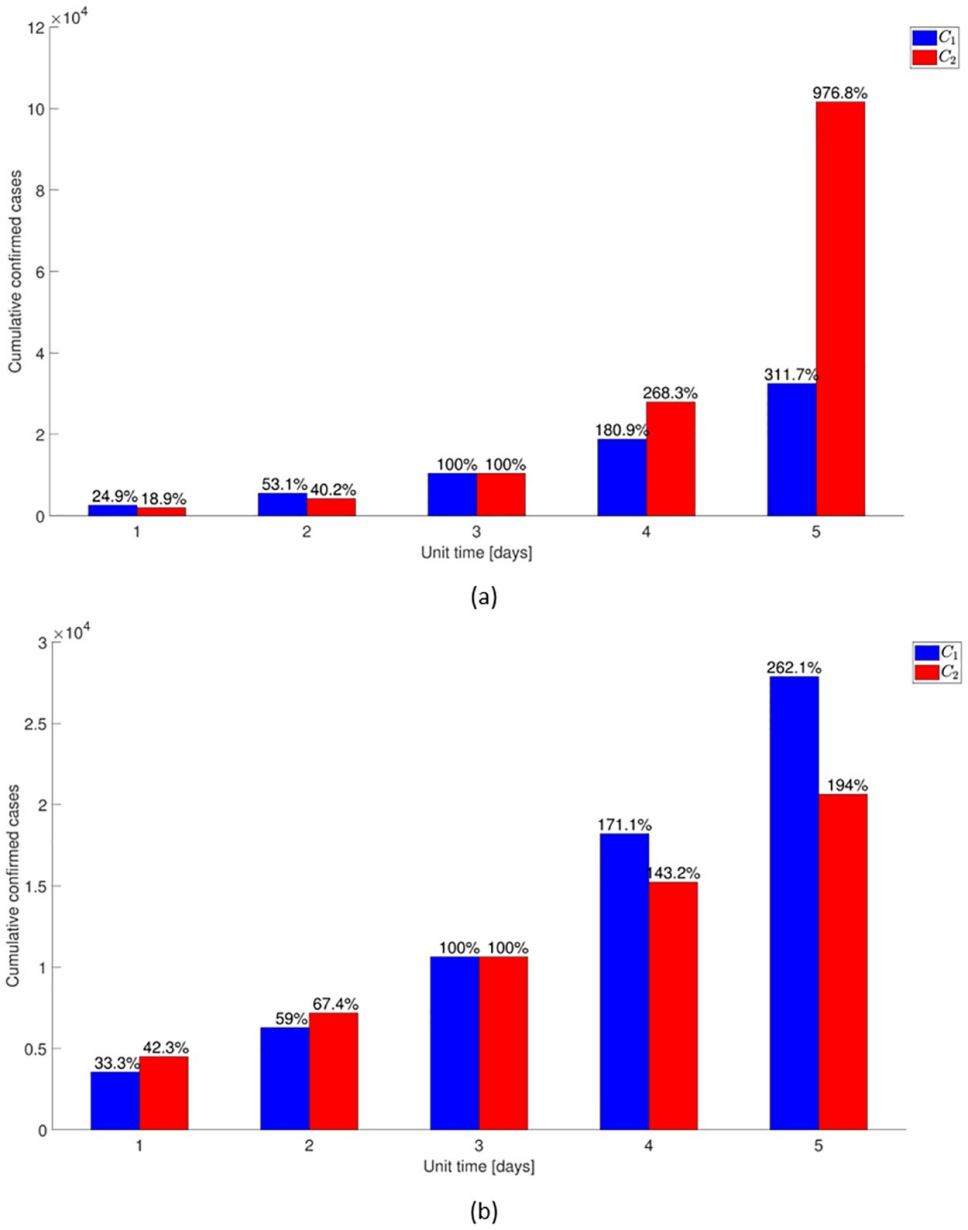
Effect of the coverage rates on the COVID-19 epidemic. The histogram shows the cumulative confirmed cases with a different quarantine rate, *C*_1_, and isolation rate, *C*_2_ under the assumption that (a) the exposed do not contribute to FOI (*ε* = 0) and (b) the exposed contribute to FOI (*ε* ≠ 0). The change in rates is represented by varying the time taken for coverage beginning on 20 February 2020 from one day to five days. Each bar in the histogram indicates the relative amount to the cumulative cases in the baseline scenario of three days.

Fig 12 visualizes how different coverage rates contribute to cumulative cases by varying the average time taken for coverage beginning on 20 February 2020, from one day to five days. In the non-infectivity of the exposed scenario (*ε* = 0), early detection and isolation are significantly more effective than quarantine because it is assumed that only the infectious can transmit the disease. However, to quarantine the exposed is as important as to isolate the infectious when we assume that the exposed also contribute to FOI (*ε* ≠ 0). S9 and S10 Figs visualize prevalence by different coverage rates. The improvement in the effect of quarantine and isolation is visible in a drastic reduction in the number of cases as the duration decreases (or coverage rate increases). Note that the change in rates for *C*_1_ and *C*_2_ in the simulation can also be interpreted as varying the coverage instead of time taken for control measures. For example, setting 60% in three days as a baseline case, 60% in two days and four days can also be considered as 75% and 50% in three days, respectively.

We also consider the number of deaths to analyze the impact of earlier (or wider) quarantine and isolation. The fatality of each age group is reported by the KDCA [12] as of 2 April 2020, as shown in Table 3. The simulated scenarios in Fig 12 combined with age-specific fatality rates, allow us to estimate the number of deaths for each age group. In Table 4, we report the total number of deaths due to COVID-19 in the baseline scenario compared to the change in coverage rates. The result is consistent with that of cumulative cases, indicating the coverage rate for isolation is much more significant than quarantine when there is no risk of infection by the exposed; in the presence of risk, the converse is true.

**Table 3:** Age-specific fatality(%) of COVID-19 reported by the KDCA as of 2 April 2020.

**Age**	0-29	30-39	40-49	50-59	60-69	70-79	80+
**Fatality**	0	0.10	0.15	0.64	1.90	7.34	18.86

**Table 4:** Effect of the coverage rate on COVID-19 death.

Period	1 day	2 days	3 days	4 days	5 days
*ε* = 0					
**Quarantine**	46.0	99.8	201.9	393.6	726.1
**Isolation**	34.5	75.0	201.9	604.6	2465.7
*ε* ≠ 0					
**Quarantine**	58.0	105.4	185.5	330.9	528.1
**Isolation**	73.7	121.3	185.5	274.0	382.6

The estimated number of deaths with different quarantine rates, C1, and isolation rate, C2 are displayed. The rates are varied by changing the time taken for coverage beginning on 20 February 2020 from one day to five days, where the baseline scenario is three days.

Then, we investigate the effects of the early implementation of quarantine and isolation using a constant coverage rate instead of piece-wise constant rates. The values for *C*_1_ and *C*_2_ after 20 February 2020 are used during the whole period to estimate the cumulative number of cases in Table 5. We find significantly fewer cumulative cases if the control measures were implemented as early as late January, 2020. The overall reduction in cases is similar, irrespective of whether we assume infectivity of the exposed.

**Table 5:** Effect of early implementation of quarantine and isolation.

Early implementation	Baseline	Quarantine	Isolation	Both
*ε* = 0				
**Cumulative cases**	10404.3	1255.9	4716.4	630.7
**Ratio to baseline [%]**	100.0	12.1	45.3	6.1
*ε* ≠ 0				
**Cumulative cases**	10633.5	1861.4	3119.7	825.5
**Ratio to baseline [%]**	100.0	17.5	29.3	7.8

The table reports the cumulative cases under the baseline scenario, early implementation of quarantine, early implementation of isolation, and early implementation of both. The last row represents the ratio of each scenario to the baseline.

## Discussion

This study has several limitations and assumptions that need to be considered. The transmission rate is estimated assuming it is proportional to the contact matrix, data for which are employed from the POLYMOD [13]. However, the current contact patterns in South Korea can be different from that in POLYMOD survey, which was conducted in European countries during a non-epidemic period. We use this source as there are no other surveys available on contact patterns in South Korea. To improve the model, future studies should consider data from new surveys conducted. To accurately predict the reduction in transmission through control measures such as social distancing, data on relevant contact patterns are required. For this reason, we analyze different scenarios which vary the reduced levels of transmission. A survey of contact patterns is underway to quantify the impact of social distance measures on contact during this current pandemic, and its data may be used in future research. Furthermore, our model does not consider asymptomatic cases, which seem to contribute to disease transmission. No reliable data sources are available, and we do not have knowledge of epidemiology to calibrate this factor.

Despite these limitations, the present findings can inform public health interventions and planning. Because it is uncertain whether the exposed are infectious, sensitivity analysis is conducted considering two scenarios: One assumes that the infectious is the only compartment which contributes to the force of infection, and the other assumes reduced risk of infection by the exposed, quarantined and isolated. In the status quo, similar results are obtained, showing that interventions are successful in bringing the reproduction number below the epidemic threshold, and that the outbreak ends around May or June after reaching the peak in early March.

Furthermore, we explore the effect of school closure and social distancing by varying the reduced levels of transmission and timing of implementation. The threshold values for the proportional factor imposed by school closure and social distancing are calculated. It has been reported that contact decreases by 25%∼75% during the break, and a recent study in the United Kingdom found a 73% reduction in the average daily number of contacts per participant. Based on this knowledge, we should be concerned about a second epidemic wave in the absence of social distancing, irrespective of whether we assume risk of infection by the exposed. However, the impact of school reopening is relatively limited compared to that of social distancing because the former applies to a single age group. We also vary the timing for the end of school closure and social distancing to find that extension of these measures by only a week causes up to a month delay in peak time.

The effect of improvement in quarantine and isolation is visible in a drastic reduction in the number of cases as the duration decreases (or coverage rate increases). In the non infectivity of the exposed scenario, early detection and isolation are significantly more effective than quarantine because only the infectious can transmit the disease. However, quarantining the exposed is as important as isolating the infectious if the exposed also contribute to FOI.

## Supporting information

S1 Appendix (PDF)

S1 Fig The result of parameter estimation for each age group (*ε* = 0). WAIFW and the rates of quarantine and isolation are fitted to the age-specific cumulative confirmed cases by assuming only the infectious contributes to the force of infection. The dotted line with circles denotes the target data, and the solid line denotes the model prediction with estimated parameters.
(TIF)

S2 Fig The newly confirmed cases for each age group predicted under the assumption that only the infectious contribute to the force of infection (*ε* = 0). (TIF)

S3 Fig The result of parameter estimation for each age group (*ε* ≠ 0). WAIFW and the rates of quarantine and isolation are fitted to the age-specific cumulative confirmed cases by assuming reduced infectivity of the exposed, quarantined, and isolated. The dotted line with circles denotes the target data, and the solid line denotes the model prediction with estimated parameters.
(TIF)

S4 Fig The newly confirmed cases for each age group predicted under the assumption that the exposed, quarantined, and isolated also contribute to the force of infection (*ε* ≠ 0). (TIF)

S5 Fig The impact of varying the timing of school reopening (*ε* = 0). The number of the infectious is displayed for each scalar factor multiplied by transmission rates when school starts on (a) 2 March 2020, (b) 6 April 2020, (c) 25 May 2020, and (d) 1 June 2020.
(TIF)

S6 Fig The impact of varying the timing of school reopening (*ε* ≠ 0). The number of the infectious is displayed for each scalar factor multiplied by transmission rates when school starts on (a) 2 March 2020, (b) 6 April 2020, (c) 25 May 2020, and (d) 1 June 2020.
(TIF)

S7 Fig The impact of varying the timing of the end of social distancing (*ε* = 0). The number of the infectious is displayed for each scalar factor multiplied by transmission rates when social distancing ends on (a) 2 March 2020, (b) 6 April 2020, (c) 25 May 2020, and (d) 1 June 2020.
(TIF)

S8 Fig The impact of varying the timing of the end of social distancing (*ε* ≠ 0). The number of the infectious is displayed for each scalar factor multiplied by transmission rates when social distancing ends on (a) 2 March 2020, (b) 6 April 2020, (c) 25 May 2020, and (d) 1 June 2020.
(TIF)

S9 Fig The impact of varying the coverage rate (*ε* = 0). The number of the infectious is displayed for each duration taken for (a) quarantine and (b) isolation at the level as in S2 Table.
(TIF)

S10 Fig The impact of varying the coverage rate (*ε* ≠ 0). The number of the infectious is displayed for each duration taken for (a) quarantine and (b) isolation at the level as in S3 Table.
(TIF)

S1 Table The parameter table used in model and result of parameter estimation. Parameters used in model are summarized in the table. The estimated *q* values for each *ε* scenario are added.
(DOCX)

S2 Table The result of parameter estimation (*ε* = 0). Piece-wise coverage rates (% in three days) of quarantine and isolation are calibrated to the age-specific cumulative confirmed cases assuming no risk of infection by the exposed.
(DOCX)

S3 Table The result of parameter estimation (*ε* ≠ 0). Piece-wise coverage rates (% in three days) of quarantine and isolation are calibrated to the age-specific cumulative confirmed cases assuming reduced risk of infection by the exposed.
(DOCX)
