# Supplementary material for: The effect of control measures on COVID-19 transmission in South Korea
Source: PLoS One. 2021 Mar 29;16(3):e0249262. doi: 10.1371/journal.pone.0249262 (PMC8006988; doi:10.1371/journal.pone.0249262)
Supplement: S1 Appendix — (PDF) [file pone.0249262.s001.pdf]

S1 Appendix. The system of equations corresponding to model diagram in Fig 1. Each compartment is stratified into 9 age groups $[0, 9], [10, 19], \cdots, [70, 79], [80, \sim)$ years, in accord with data provided by KDCA. In the following model equations, i-th age group is denoted by the subscript i.

$$\begin{aligned}
\frac{dS_i}{dt} &= -\lambda_i(t)S_i - \delta S_i + \tau Q_{S_i} \\
\frac{dE_i}{dt} &= \lambda_i(t)S_i - fE_i - C_1(t)E_i \\
\frac{dI_i}{dt} &= fE_i - \gamma I_i - C_2(t)I_i \\
\frac{dR_i}{dt} &= \gamma I_i + \gamma Q_{I_i} \\
\frac{dQ_{S_i}}{dt} &= \delta S_i - \tau Q_{S_i} \\
\frac{dQ_{E_i}}{dt} &= C_1(t)E_i - fQ_{E_i} \\
\frac{dQ_{I_i}}{dt} &= fQ_{E_i} + C_2(t)I_i - \gamma Q_{I_i}
\end{aligned} \tag{6}$$
