# Supplementary figures and images for: The effect of control measures on COVID-19 transmission in South Korea
Source: PLoS One. 2021 Mar 29;16(3):e0249262. doi: 10.1371/journal.pone.0249262 (PMC8006988; doi:10.1371/journal.pone.0249262)

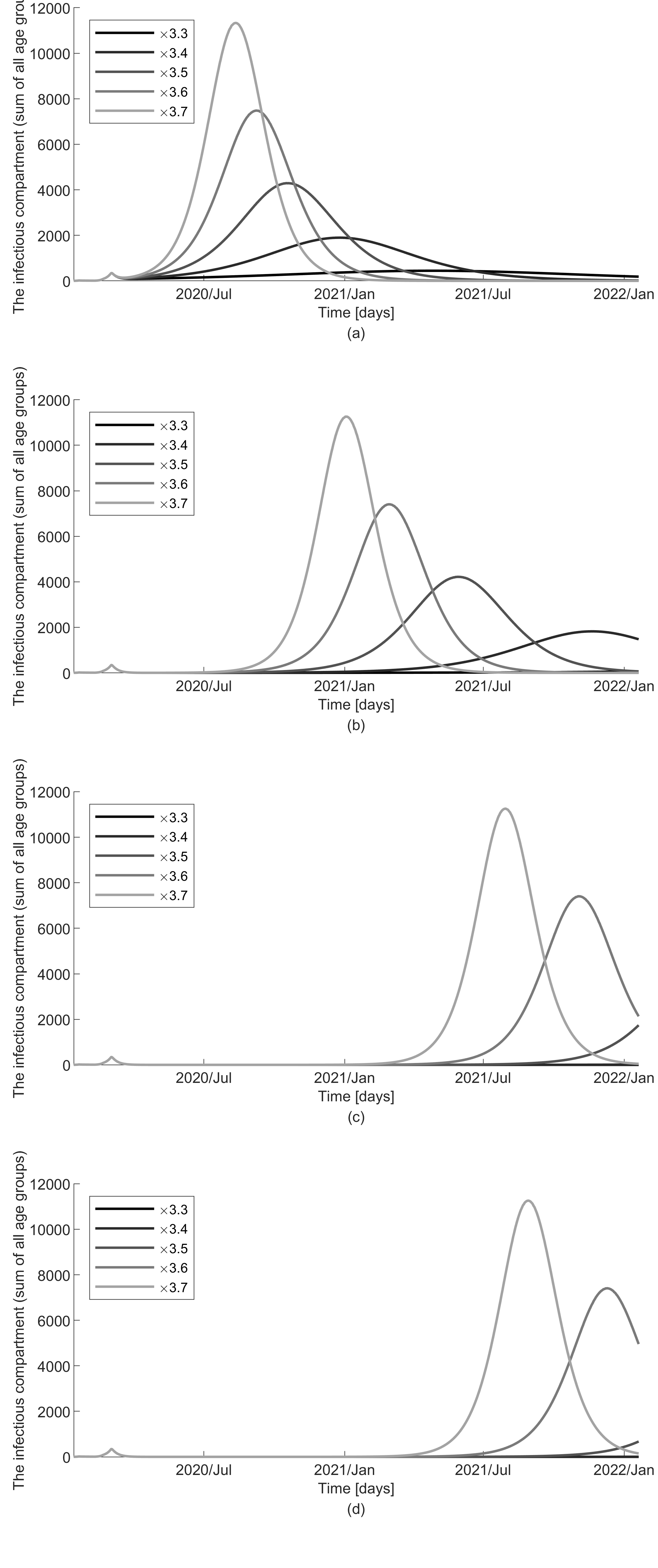

Supplement: S5 Fig — The number of the infectious is displayed for each scalar factor multiplied by transmission rates when school starts on (a) 2 March 2020, (b) 6 April 2020, (c) 25 May 2020, and (d) 1 June 2020. (TIF) [file pone.0249262.s006.tif]

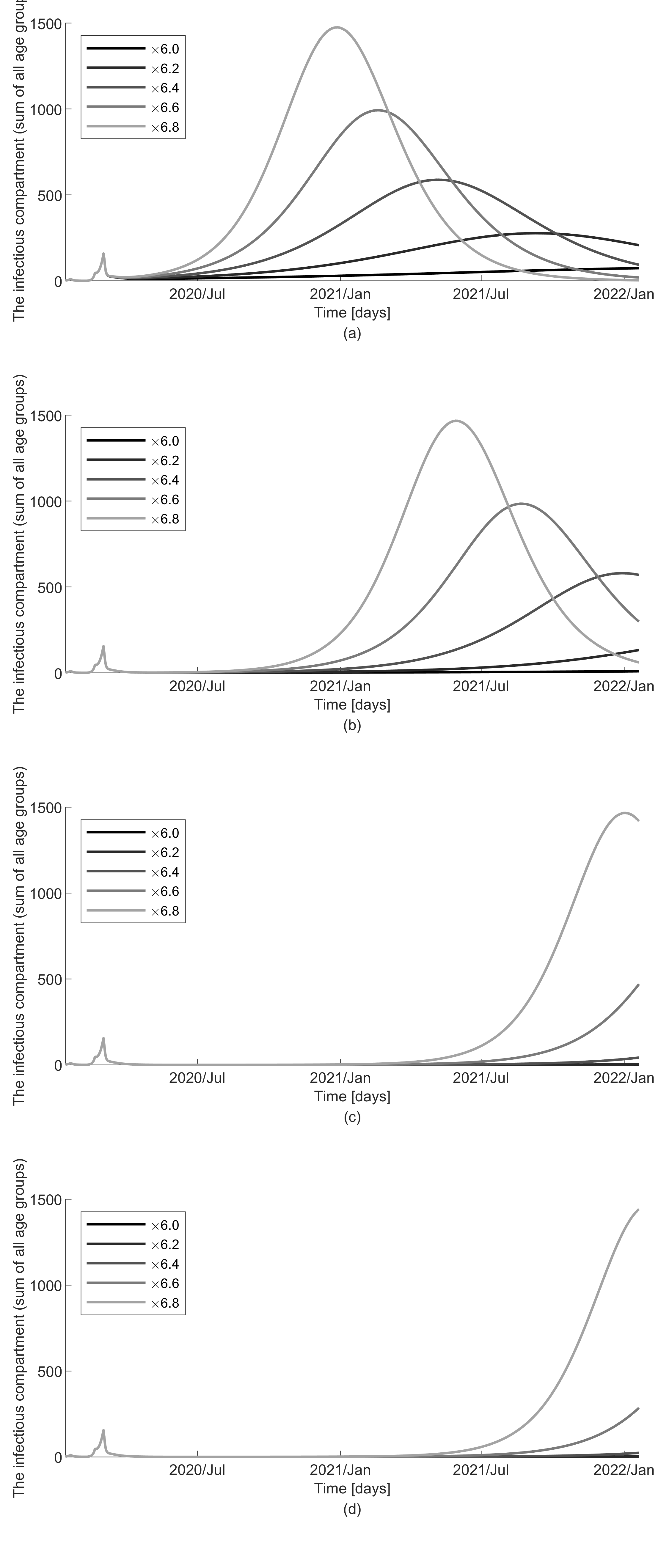

Supplement: S6 Fig — The number of the infectious is displayed for each scalar factor multiplied by transmission rates when school starts on (a) 2 March 2020, (b) 6 April 2020, (c) 25 May 2020, and (d) 1 June 2020. (TIF) [file pone.0249262.s007.tif]

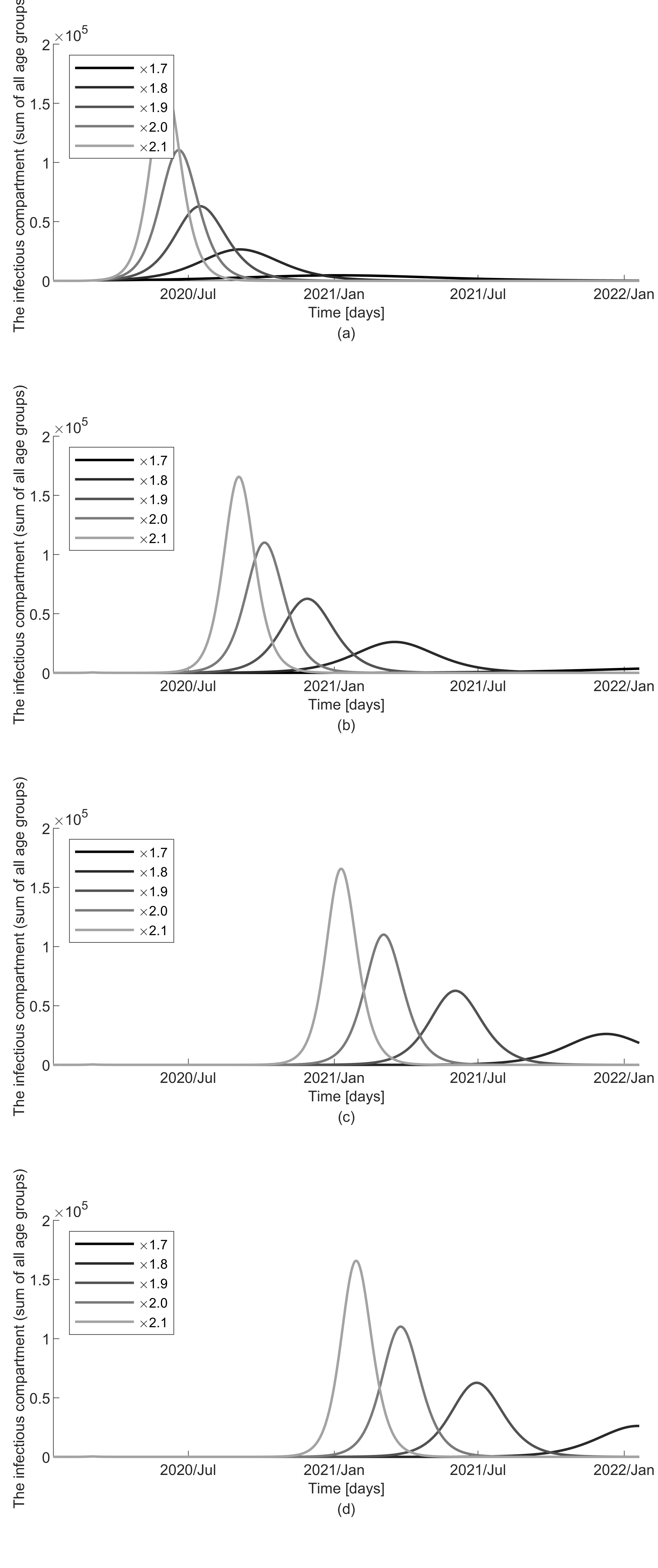

Supplement: S7 Fig — The number of the infectious is displayed for each scalar factor multiplied by transmission rates when social distancing ends on (a) 2 March 2020, (b) 6 April 2020, (c) 25 May 2020, and (d) 1 June 2020. (TIF) [file pone.0249262.s008.tif]

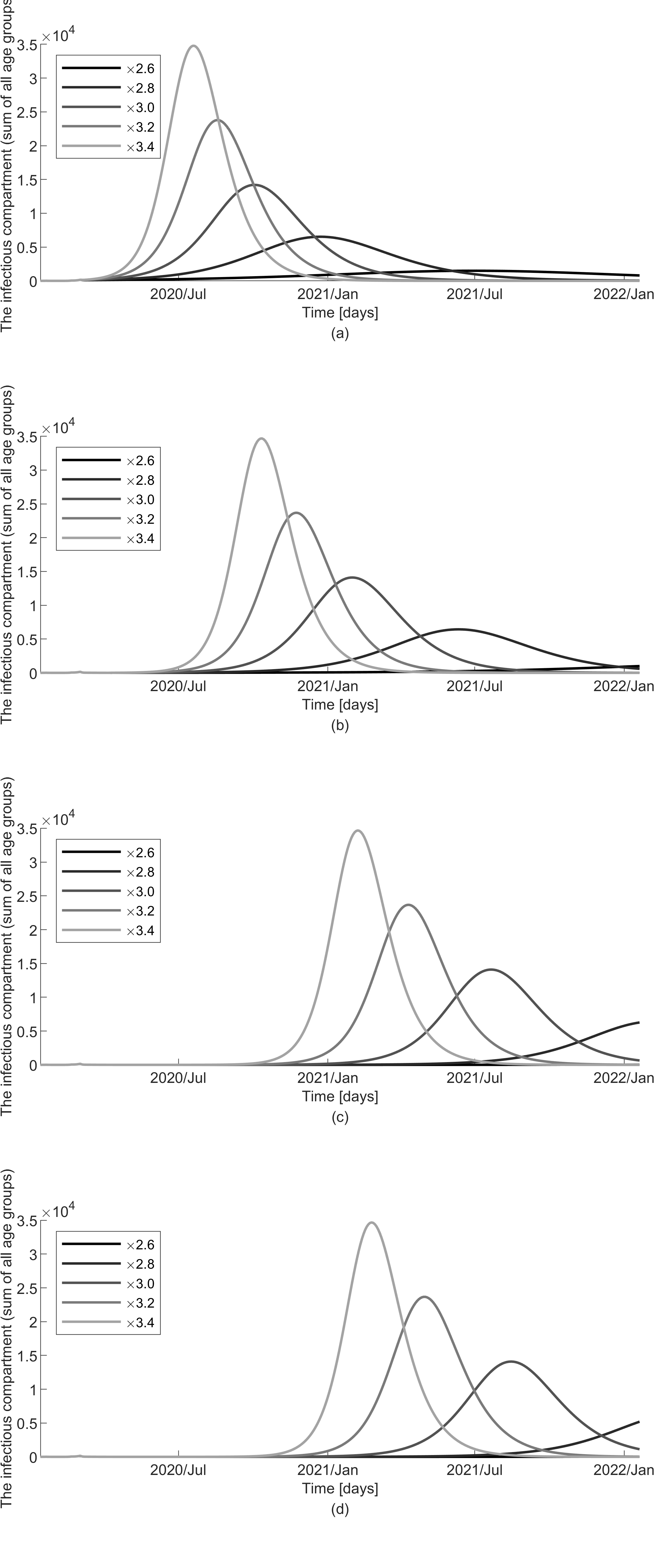

Supplement: S8 Fig — The number of the infectious is displayed for each scalar factor multiplied by transmission rates when social distancing ends on (a) 2 March 2020, (b) 6 April 2020, (c) 25 May 2020, and (d) 1 June 2020. (TIF) [file pone.0249262.s009.tif]

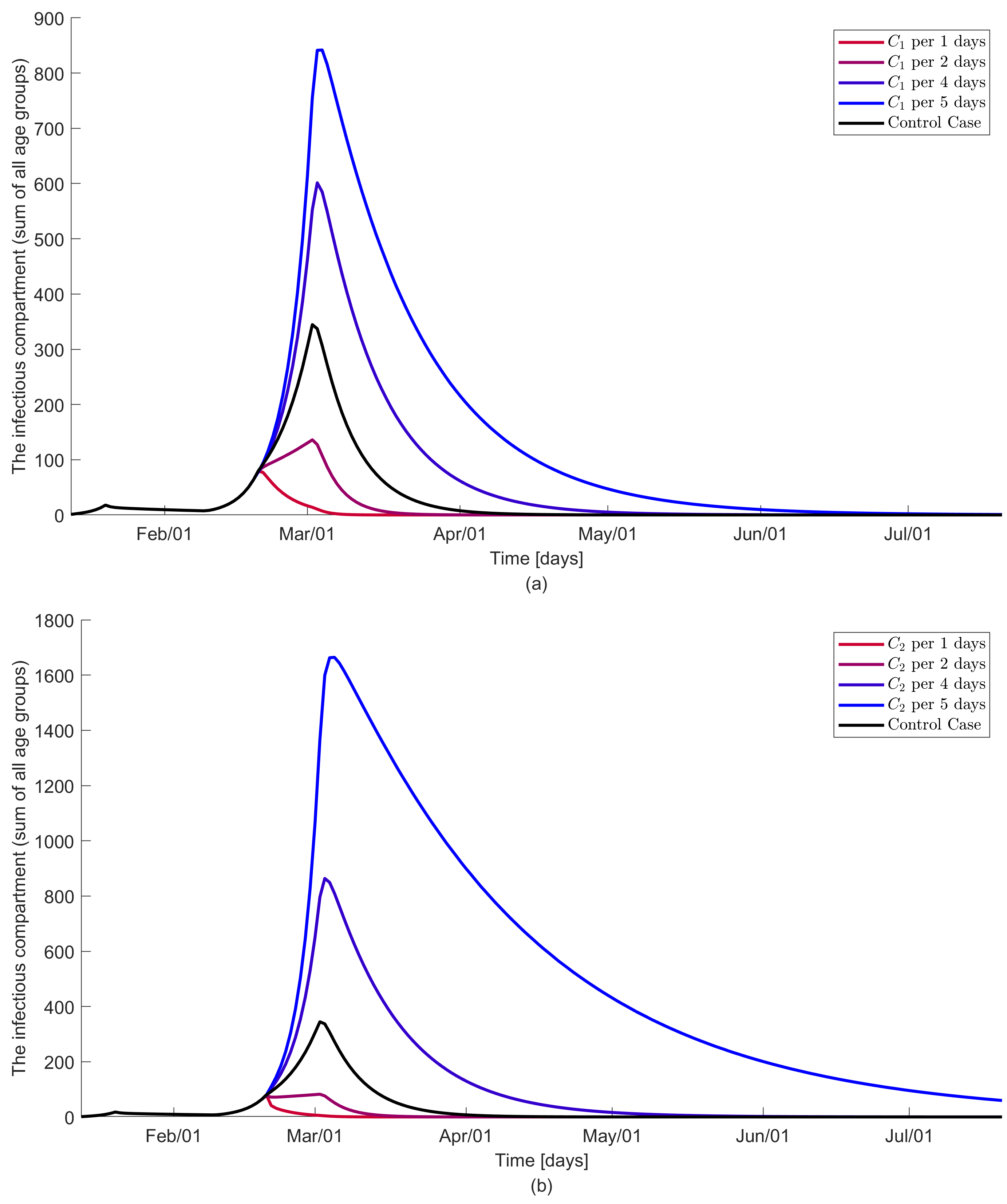

Supplement: S9 Fig — The number of the infectious is displayed for each duration taken for (a) quarantine and (b) isolation at the level as in S2 Table. (TIF) [file pone.0249262.s010.tif]

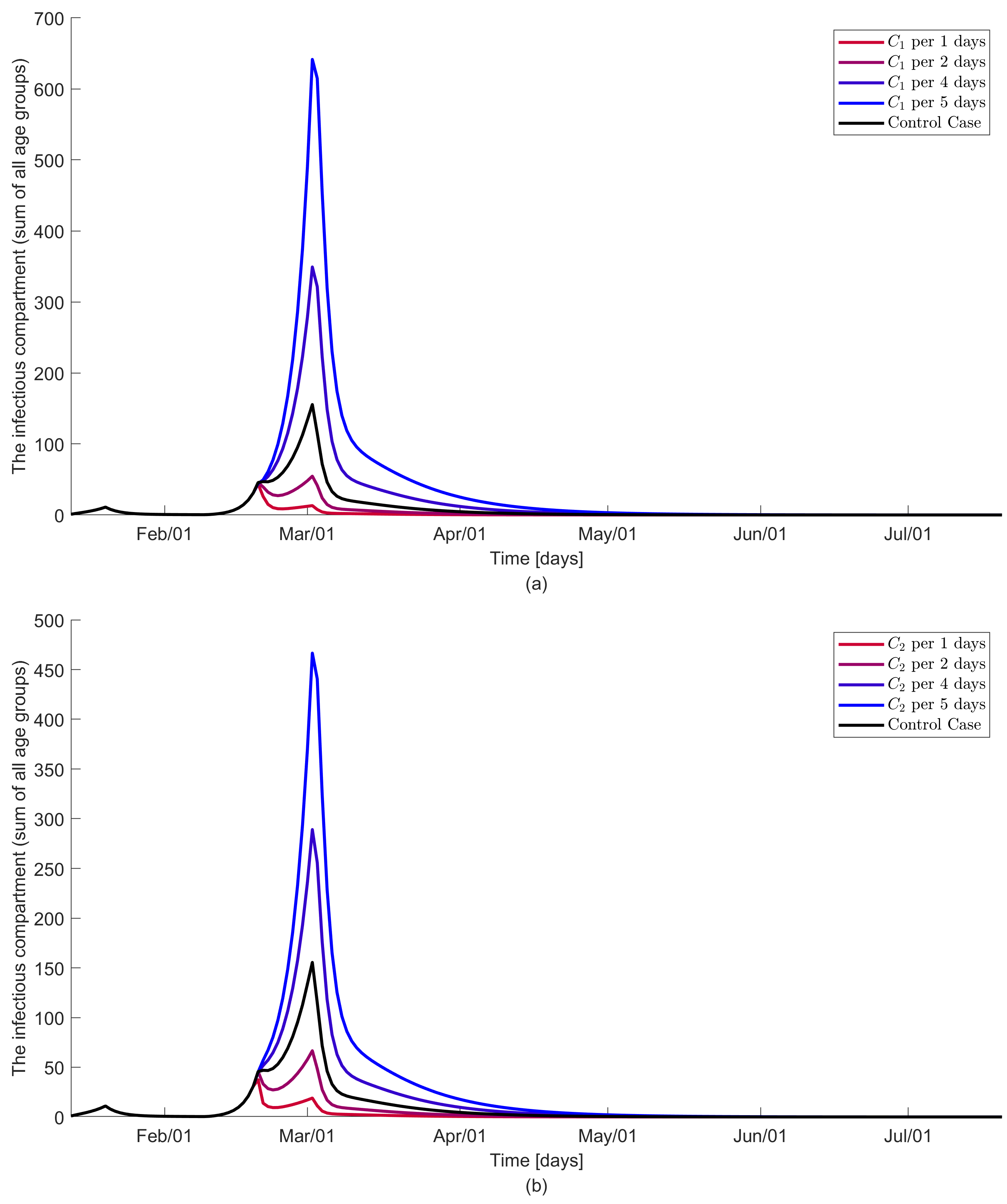

Supplement: S10 Fig — The number of the infectious is displayed for each duration taken for (a) quarantine and (b) isolation at the level as in S3 Table. (TIF) [file pone.0249262.s011.tif]
