# Supplementary material for: The effect of control measures on COVID-19 transmission in South Korea
Source: PLoS One. 2021 Mar 29;16(3):e0249262. doi: 10.1371/journal.pone.0249262 (PMC8006988; doi:10.1371/journal.pone.0249262)
Supplement: S1 Table — Parameters used in model are summarized in the table. The estimated q values for each ε scenario are added. (DOCX) [file pone.0249262.s012.docx]

**S1 Table.** Parameters being used in simulation.

| **Parameters** | **Symbol** | **Value** |
| --- | --- | --- |
| Ineffective quarantine rate | $\delta$ | $7.2150\times{10}^{-4}$  Calculated for asymptotic ratio $S:Q_{S}=99:1$  (Assumed) |
| Quarantine duration [days] | $1/\tau$ | 14 |
| Infectivity of the exposed | $\varepsilon_{E}$ | 0 or 0.1 (Scenario) |
| Infectivity of the quarantined | $\varepsilon_{Q_{E}}$ | 0 or 0.01 (Scenario) |
| Infectivity of the isolated | $\varepsilon_{Q_{I}}$ | 0 or 0.05 (Scenario) |
| Exposed period [days] | $1/f$ | 5.2 |
| Quarantine proportion [%/3days] | $C_{1}$ | Estimated (Changed at 20 Feb 2020) |
| Isolation proportion [%/3days] | $C_{2}$ | Estimated (Changed at 20 Feb 2020) |
| Infectious period [days] | $1/\gamma$ | 7 |
| WAIFW matrix | $W$ | Calculated from the estimated $q$ values below |
| Transmission rates from age 0-9  Transmission rates from age 10-19  Transmission rates from age 20-29  Transmission rates from age 30-39  Transmission rates from age 40-49  Transmission rates from age 50-59  Transmission rates from age 60-69  Transmission rates from age 70-79  Transmission rates from age 80+ | $q_{0}$  $q_{1}$  $q_{2}$  $q_{3}$  $q_{4}$  $q_{5}$  $q_{6}$  $q_{7}$  $q_{8}$ | Estimated for each interval  (Changed at 9 Feb 2020 and 2 Mar 2020) |
| Contact rate from $j$-th age group to $i$-th age group | $c_{ij}$ | Calculated from the POLYMOD survey |

Detail estimated values for $q$ when $\varepsilon=0$.

|  | **Until 8 Feb 2020** | **9 Feb 2020 ~ 1 Mar 2020** | **From 2 Mar 2020** |
| --- | --- | --- | --- |
| $q_{0}$ | 0.0801 | 0.1040 | 0.0801 |
| $q_{1}$ | 0.1695 | 0.3323 | 0.1695 |
| $q_{2}$ | 0.4527 | 1.1226 | 0.4527 |
| $q_{3}$ | 0.2209 | 0.6037 | 0.2209 |
| $q_{4}$ | 0.2696 | 0.7303 | 0.2696 |
| $q_{5}$ | 0.3903 | 0.9870 | 0.3903 |
| $q_{6}$ | 0.5488 | 1.4153 | 0.5488 |
| $q_{7}$ | 0.9562 | 1.3677 | 0.9562 |
| $q_{8}$ | 1.3167 | 2.2443 | 1.3167 |

Detail estimated values for $q$ when $\varepsilon\neq0$.

|  | **Until 8 Feb 2020** | **9 Feb 2020 ~ 1 Mar 2020** | **From 2 Mar 2020** |
| --- | --- | --- | --- |
| $q_{0}$ | 0.0602 | 0.1099 | 0.0602 |
| $q_{1}$ | 0.0744 | 0.3946 | 0.0744 |
| $q_{2}$ | 0.2426 | 1.2787 | 0.2426 |
| $q_{3}$ | 0.1255 | 0.7052 | 0.1255 |
| $q_{4}$ | 0.1236 | 0.8279 | 0.1236 |
| $q_{5}$ | 0.1566 | 1.1917 | 0.1566 |
| $q_{6}$ | 0.3383 | 1.5562 | 0.3383 |
| $q_{7}$ | 0.5272 | 1.7317 | 0.5272 |
| $q_{8}$ | 1.0400 | 1.5547 | 1.0400 |
