# Supplementary material for: The effect of control measures on COVID-19 transmission in South Korea
Source: PLoS One. 2021 Mar 29;16(3):e0249262. doi: 10.1371/journal.pone.0249262 (PMC8006988; doi:10.1371/journal.pone.0249262)
Supplement: S2 Table — Piece-wise coverage rates (% in three days) of quarantine and isolation are calibrated to the age-specific cumulative confirmed cases assuming no risk of infection by the exposed. (DOCX) [file pone.0249262.s013.docx]

**S2 Table.** The result of parameter estimation ($\varepsilon=0$). Piece-wise coverage rates (% in three days) of quarantine and isolation are calibrated to the age-specific cumulative confirmed cases assuming no risk of infection by the exposed.

| **Time** | **Before 20 February 2020** | **After 20 February 2020** |
| --- | --- | --- |
| **Quarantine rate** | 43.5 | 60.7 |
| **Isolation rate** | 82.4 | 85.8 |
