# Supplementary material for: The effect of control measures on COVID-19 transmission in South Korea
Source: PLoS One. 2021 Mar 29;16(3):e0249262. doi: 10.1371/journal.pone.0249262 (PMC8006988; doi:10.1371/journal.pone.0249262)
Supplement: S3 Table — Piece-wise coverage rates (% in three days) of quarantine and isolation are calibrated to the age-specific cumulative confirmed cases assuming reduced risk of infection by the exposed. (DOCX) [file pone.0249262.s014.docx]

**S3 Table.** The result of parameter estimation ($\varepsilon\neq0$). Piece-wise coverage rates (% in three days) of quarantine and isolation are calibrated to the age-specific cumulative confirmed cases assuming reduced risk of infection by the exposed.

| **Time** | **Before 20 February 2020** | **After 20 February 2020** |
| --- | --- | --- |
| **Quarantine rate** | 79.0 | 94.6 |
| **Isolation rate** | 81.7 | 94.7 |
